# Mortality of Head and Neck Cancer in China From 1990 to 2019: A Secondary Data Analysis

**DOI:** 10.1002/cnr2.70116

**Published:** 2025-03-12

**Authors:** Zhengxin Zhu, Jiasheng Yuan, Li Fu, Wanqing Zhang, Songtao Liu, Yuehui Liu

**Affiliations:** ^1^ Department of Otorhinolaryngology Head and Neck Surgery, the Second Affiliated Hospital, Jiangxi Medical College Nanchang University Nanchang Jiangxi China

**Keywords:** age–period–cohort analysis, head and neck cancer, joinpoint regression, mortality, trend

## Abstract

**Background:**

The rising global incidence of head and neck cancer imposes a growing burden on health systems. However, comprehensive analysis of mortality trends, particularly age, period, and cohort effects, remains limited.

**Objective:**

This study aims to evaluate head and neck cancer mortality trends in China from 1990 to 2019, with a focus on age, period, and cohort effects.

**Methods:**

A secondary data analysis was conducted using data from the Global Burden of Disease Study, focusing on the Chinese population aged 20 years and older. The cancers examined included those of the lip–oral cavity, nasopharynx, other pharynx, larynx, and thyroid. Mortality data, including death numbers and age‐standardized rates, were analyzed using joinpoint regression and age–period–cohort analysis to identify trends.

**Results:**

The study revealed that from 1990 to 2019, the age‐standardized mortality rate for head and neck cancer in China decreased more significantly than the global average. Although the overall trend in China showed a decrease, there were sporadic increases, especially among males. In contrast, females exhibited a more consistent decline. The age–period–cohort analysis demonstrated increasing mortality with age, decreasing mortality over successive periods, and fluctuating cohort effects, with a marked decrease for cohorts born after 1930.

**Conclusion:**

Overall, the mortality rate for head and neck cancer in China is declining, with age being a significant risk factor for mortality, and earlier‐born cohorts facing higher risks. Continuous monitoring is essential to understand the impact of evolving clinical practice guidelines on the mortality of head and neck cancer.

## 1 Introduction

Head and neck cancer (HNC) ranks as the seventh most common cancer worldwide [1], comprising subtypes like lip–oral cavity cancer (LOC), nasopharynx cancer (NPC), other pharynx cancer (OPC), larynx cancer (LC), and thyroid cancer (TC) [2]. These subtypes pose distinct challenges in otolaryngology head and neck surgery. In 2020, over 930 000 new HNC cases were diagnosed globally, with China accounting for about 15% [3].

Subtle early symptoms complicate HNC detection, often resulting in late‐stage diagnosis [4, 5]. Despite advances in treatment combining surgery, radiotherapy, and chemotherapy, the 5‐year survival rate remains around 50% [6]. HNC also imposes a significant economic burden on the healthcare system. For instance, in the United States, the economic burden rose from approximately $976 million [7] in 2001 to $3.64 billion [8] in 2010. Therefore, understanding mortality trends is crucial for evaluating public health interventions and formulating strategies to reduce this burden.

Current research on HNC mortality trends is limited, often confined to age‐specific incidence or mortality rates, lacking detailed analysis of age, period, and cohort interactions. This study aims to bridge this gap using the Joinpoint Regression (JPR) model and age–period–cohort analysis. JPR identifies “joinpoints” in data to depict trend shifts [9], whereas age–period–cohort distinguishes mortality risks across generations and time periods [10, 11], reflecting public health policy impacts and healthcare advancements.

Utilizing data from the Global Burden of Disease (GBD) study 2019, this research employs JPR and age–period–cohort analyses to examine HNC mortality trends in China comprehensively. The findings aim to inform academic discourse and guide policymakers and healthcare professionals in mitigating HNC mortality.

## 2 Methods

### 2.1 Data Sources

Death numbers and age‐standardized mortality rate (ASMR) for HNC in China and globally from 1990 to 2019, as well as the corresponding age‐specific population data, were obtained from the GBD database (available from https://vizhub.healthdata.org/gbd‐results/) [12], provided by the Institute for Health Metrics and Evaluation at the University of Washington. The GBD database, which assessed the disease burden caused by 369 diseases and injuries across 204 countries and regions, utilized multiple global data sources and specific models for its calculations [13]. Mortality data, gathered from vital registration, verbal autopsy reports, and surveillance sources, were integrated into the GBD cause‐of‐death database [14]. Subsequently, these data underwent Bayesian meta‐regression analysis using the DisMod‐MR 2.19 tool, which adjusts for bias and generates disease estimates with 95% uncertainty intervals (UIs) [13, 15]. This study adhered to the Strengthening the Reporting of Observational Studies in Epidemiology (STROBE) reporting guideline [16].

Previous publications described the exact GBD methods in greater detail [13, 17]. This research included five types of HNC: LOC, NPC, OPC, LC, and TC. The International Classification of Diseases 10th Revision codes (ICD‐10) were as follows: LOC (C00–C08.9, D10.0–D10.5, and D11–D11.9), NPC (C11–C11.9 and D10.6), OPC (C09–C10.9, C12–C13.9, and D10.7), LC (C32–C32.9, D02.0, D14.1, and D38.0), and TC (C73–C73.9, D09.3, D09.8, D34–D34.9, and D44.0) [18]. The data were age‐standardized using the GBD standard population to ensure comparability across different populations. Considering the comparatively low mortality rates of HNC among individuals under 20 years old in China, and the relatively small population percentage over 85 years old, coupled with the high prevalence of comorbidities in this latter age group, interventions targeting these age brackets tend to be less efficacious. Consequently, this study excluded data pertaining to individuals below 20 years of age and consolidated data for those over 85 years old. Ultimately, 14 age groups were included in the analysis. For trend analysis, the ASMRs for HNC were log‐transformed to meet the assumptions of the JPR model.

### 2.2 JPR Analysis

The JPR model [9], developed by the National Cancer Institute, was utilized to analyze the temporal trends of ASMR for HNC from 1990 to 2019. This model segments the long‐term trend of the disease into distinct phases based on its distribution characteristics, fitting a log‐linear model to the data [19]. The intersections of these segments, termed joinpoints, are identified using the Monte Carlo permutation test method [9], which determines their location, number, and statistical significance. The model's equation is expressed as: *E*(*y|x*) = *β*_0_ + *β*_1_ *x* + *δ*_1_(*x*−*τ*_*1*_)^+^+⋯ + *δ*_*k*_(*x*−*τ*_*k*_)^+^ [9, 20]. Here, *y* denotes the HNC mortality rate, *x* represents the observation year, *β* is the constant term, *δ* are the regression coefficients for each segment function (*i* = 1, 2, 3…*k*), and *τ* represents the joinpoints (*i* = 1, 2, 3…*k*). The term (*x*−*τ*_*k*_)^+^ is defined as (*x*−*τ*_*k*_) when (*x*−*τ*_*k*_) ≥ 0, and 0 otherwise.

The analysis estimated the annual percentage change (APC) for each segment and the average APC (AAPC) across the entire study period. APC and AAPC were presented alongside their 95% confidence intervals (CIs) to evaluate the magnitude and direction of the trends.

### 2.3 Age–Period–Cohort Analysis

The age–period–cohort analysis was used to assess the influence of age, period, and cohort effects on the mortality rate of HNC. Leveraging the Poisson distribution, this method enhances traditional descriptive analysis by decomposing the analysis variable into age, period, and cohort dimensions [21, 22]. It serves as a pivotal tool in demography and epidemiology for analyzing trends in chronic disease incidence and mortality rates and for forecasting future disease burden shifts [23]. Our study concentrated on estimating net drift, longitudinal age curve, period, and cohort rate ratios (RRs). Net drift reflects the APC in expected age‐adjusted rates, whereas the longitudinal age curve represents age‐specific rates adjusted for cohort and period effects. Period and cohort RRs are the relative risks for each period or cohort, adjusted for age and nonlinear effects, compared to a reference period or cohort [24, 25]. The Wald *χ* ^2^ test was applied to determine the significance of age, period, and cohort effects.

### 2.4 Statistical Analysis

Data analysis was conducted from January 10 to March 15, 2024. The Joinpoint Regression Program software (Version 5.0.2, May 2023) was utilized to fit the JPR model to the ASMR for male and female HNC populations. The JPR model identifies shifts in the ASMR trends over time, with an APC less than 0 indicating a decreasing mortality rate, an APC greater than 0 indicating an increase, and an APC equal to AAPC suggesting a consistent trend. The age–period–cohort web tool [26] was employed for age–period–cohort analysis [24], and R software (Version 4.3.1) facilitated data visualization. Subjects were categorized into 14 age groups at 5‐year intervals. To meet the age–period–cohort model's requirements, the study period was divided into six 5‐year intervals, and the birth cohorts were segmented into 19 groups spanning from 1905 to 1995. All statistical tests were two‐sided, with a significance level set at *α* = 0.05.

### 2.5 Ethical Considerations

The present study constitutes a secondary analysis of de‐identified data, which is publicly accessible from the GBD study 2019, approved by the Institutional Review Board of the University of Washington. The original data collection was performed with informed consent or with a waiver from the Institutional Review Board. In line with the data use agreement from the Institute for Health Metrics and Evaluation, no additional ethical approval was necessitated for this analysis.

## 3 Results

### 3.1 Trend

The estimated number of deaths and ASMR between 1990 and 2019 in China, stratified by sex, are demonstrated in Table 1, compared to the global level. In 2019, the number of deaths from HNC was 84 385.4 (95% UI: 69 996.1, 100 582) in China and 554 146.3 (95% UI: 506 478.1, 603 296.3) cases globally, respectively. Meanwhile, ASMRs were 4.27 (95% UI: 3.56, 5.06) in China and 6.74 (95% UI: 6.16, 7.33) globally. Between 1990 and 2019, the ASMR of HNC in China decreased by 28.87% (95% CI: −31.54%, −26.29%), whereas it decreased by 12.86% (95% CI: −15%, −11.45%) globally. When analyzed by sex, the drop of ASMR in China was higher in females than in males, in contrast to the global level. Examining specific cancer types, the ASMR for NPC, OPC, LC, and TC all showed a declining trend, whereas LOC exhibited an increasing trend. Interestingly, in the cases of LOC and TC, the ASMR trends for males and females were opposite, with a significant upward trend for males and a downward trend for females.

**TABLE 1:** Death population and age‐standardized mortality rates of head and neck cancer from 1990 to 2019.

Variable		Both sexes					Male					Female				
		1990		2019		1990–2019	1990		2019		1990–2019	1990		2019		1990–2019
		Death numbers, *n* (95% UI)	ASMR per 100 000, *n* (95% UI)	Death numbers, *n* (95% UI)	ASMR per 100 000, *n* (95% UI)	% Change in ASMR, *n*% (95% CI)	Death numbers, *n* (95% UI)	ASMR per 100 000, *n* (95% UI)	Death numbers, *n* (95% UI)	ASMR per 100 000, *n* (95% UI)	% Change in ASMR, *n*% (95% CI)	Death numbers, *n* (95% UI)	ASMR per 100 000, *n* (95% UI)	Death numbers, *n* (95% UI)	ASMR per 100 000, n (95% UI)	% Change in ASMR, *n*% (95% CI)
Global	Overall	311970.9 (292176.1–334191.3)	7.73 (7.24–8.27)	554146.3 (506478.1–603296.3)	6.74 (6.16–7.33)	−12.86 (−15 to −11.45)	224811.9 (207542.6–243444.4)	11.97 (11.07–12.93)	394991.5 (356193.4–435828.7)	10.18 (9.19–11.23)	−14.94 (−17.03 to −13.15)	87 159 (78155.6–96 235)	4.08 (3.67–4.5)	159154.8 (141359.2–177939.2)	3.67 (3.26–4.1)	−10.19 (−11.21 to −8.91)
	LOC	96627.5 (90592.3–103050.1)	2.44 (2.28–2.6)	199397.6 (181651.5–218058.6)	2.44 (2.22–2.66)	0.03 (−2.51 to 2.59)	66994.6 (61237.5–73049.9)	3.63 (3.32–3.95)	131560.7 (117700.3–145 460)	3.42 (3.06–3.77)	−5.89 (−7.77 to −4.39)	29632.9 (27302.1–31843.6)	1.41 (1.3–1.52)	67836.8 (60782.4–75652.9)	1.56 (1.4–1.74)	10.37 (7.31 to 14.67)
	NPC	53458.8 (48874.8–57905.9)	1.26 (1.15–1.36)	71610.5 (65 442–77624.6)	0.86 (0.79–0.93)	−31.35 (−31.48 to −31.18)	35111.8 (31341.4–38915.7)	1.72 (1.55–1.9)	51 223 (45965.2–56950.9)	1.28 (1.15–1.43)	−25.58 (−25.42 to −25.03)	18 347 (15568.6–20725.5)	0.83 (0.71–0.94)	20387.5 (18 156–22777.4)	0.47 (0.42–0.53)	−42.93 (−40.16 to −43.42)
	OPC	51459.6 (47973.6–56456.8)	1.25 (1.17–1.37)	114206.7 (103153.6–126039.4)	1.37 (1.24–1.51)	9.55 (5.96 to 10.03)	39179.9 (35993.1–43329.6)	2.04 (1.86–2.25)	88017.2 (77951.5–98653.6)	2.23 (1.98–2.5)	9.66 (6.12 to 11.19)	12279.6 (10602.1–14135.3)	0.57 (0.49–0.65)	26189.5 (22518.4–30492.3)	0.6 (0.52–0.7)	6.07 (5.29 to 7.57)
	LC	87458.6 (83181.6–91550.9)	2.19 (2.08–2.29)	123355.6 (114941.4–132798.4)	1.49 (1.39–1.61)	−31.64 (−33.07 to −29.74)	75929.9 (72031.5–79650.9)	4.15 (3.94–4.35)	105555.5 (97754.9–114522.1)	2.74 (2.54–2.98)	−33.82 (−35.66 to −31.54)	11528.7 (10662.9–12280.3)	0.54 (0.5–0.58)	17800.1 (16 184–19687.9)	0.41 (0.37–0.45)	−24.78 (−26.02 to −21.89)
	TC	22966.4 (21553.8–25227.7)	0.6 (0.56–0.66)	45 576 (41289.6–48775.3)	0.57 (0.51–0.61)	−4.91 (−8.7 to −7.49)	7595.7 (6939.1–8498.4)	0.43 (0.4–0.48)	18635.2 (16821.6–20242.1)	0.51 (0.46–0.55)	16.83 (14.37 to 14.48)	15370.7 (14 020–17250.3)	0.73 (0.66–0.82)	26940.8 (23718.4–29328.7)	0.62 (0.55–0.68)	−14.65 (−17.85 to −17.19)
China	Overall	51778.4 (44485.3–59442.9)	6.01 (5.2–6.87)	84385.4 (69996.1–100 582)	4.27 (3.56–5.06)	−28.87 (−31.54 to −26.29)	34388.2 (28133.9–40858.4)	8.36 (6.95–9.83)	64603.5 (50988.9–80 075)	6.98 (5.58–8.55)	−16.54 (−19.71 to −13.02)	17390.2 (14127.1–20897.8)	3.99 (3.26–4.77)	19781.9 (15686.3–24246.9)	1.96 (1.55–2.39)	−50.96 (−52.32 to −49.84)
	LOC	7403.2 (6437.6–8357.9)	0.92 (0.81–1.03)	22641.7 (18908.1–27 077)	1.16 (0.98–1.38)	25.79 (20.37 to 33.29)	4570.7 (3724.2–5446.7)	1.23 (1.02–1.44)	17 608 (14093.6–21897.3)	1.95 (1.59–2.4)	59.34 (55.89 to 66.58)	2832.6 (2355.4–3331.1)	0.69 (0.57–0.8)	5033.8 (4058.5–6116.9)	0.5 (0.41–0.61)	−26.64 (−28.93 to −23.8)
	NPC	26571.1 (22 772–30278.4)	2.9 (2.51–3.3)	28659.5 (23 780–34066.1)	1.43 (1.19–1.69)	−50.8 (−52.48 to −48.77)	17427.8 (14301.6–20733.5)	3.86 (3.21–4.55)	21349.2 (16674.8–26466.4)	2.2 (1.74–2.7)	−43.11 (−45.78 to −40.73)	9143.3 (7281–11019.7)	2.01 (1.61–2.42)	7310.3 (5704.2–9036.1)	0.72 (0.56–0.89)	−64.37 (−65.14 to −63.43)
	OPC	2989.9 (2565.2–3438.1)	0.36 (0.31–0.41)	5590.4 (4533.9–6731)	0.28 (0.23–0.33)	−22.49 (−27.37 to −18.76)	2258.1 (1859.8–2666.9)	0.58 (0.49–0.69)	4597.1 (3546.7–5738.6)	0.49 (0.38–0.6)	−16.85 (−22.54 to −12.44)	731.8 (602.6–866.1)	0.17 (0.14–0.2)	993.3 (789.7–1209.7)	0.1 (0.08–0.12)	−43.28 (−46.18 to −41.27)
	LC	11495.4 (9849–13235.3)	1.4 (1.21–1.6)	20254.7 (16762.2–24231.6)	1.02 (0.85–1.21)	−27.48 (−29.88 to −24.4)	8901.9 (7244.4–10487.1)	2.34 (1.94–2.72)	16837.2 (13495.5–20731.4)	1.82 (1.47–2.21)	−22.13 (−24.15 to −18.67)	2593.5 (2177.5–3000.2)	0.62 (0.52–0.71)	3417.5 (2738.7–4125.3)	0.34 (0.27–0.4)	−46.03 (−48.66 to −43.42)
	TC	3318.8 (2861.5–4133.2)	0.42 (0.37–0.53)	7239.1 (6011.9–8476.3)	0.39 (0.32–0.45)	−7.63 (−12.52 to −14.04)	1229.8 (1003.8–1524.2)	0.35 (0.29–0.43)	4212.1 (3178.4–5241.3)	0.52 (0.4–0.64)	48.65 (37.53 to 49.03)	2089 (1710.7–2680.7)	0.5 (0.41–0.63)	3027 (2395.3–3758.8)	0.3 (0.24–0.38)	−39.01 (−41.54 to −40.8)

Abbreviations: ASMR, age‐standardized mortality rate; CI, confidence interval; LC, larynx cancer; LOC, lip–oral cavity cancer; NPC, nasopharynx cancer; OPC, other pharynx cancer; TC, thyroid cancer; UI, uncertainty interval.

### 3.2 JPR Analysis

The temporal trends in ASMR for HNCs in China are presented in Table 2. Downward trends from 1990 to 2019 are shown (AAPC = −1.21%). The ASMR of HNC significantly declined initially (APC_1990–1997_ = −2.01%, APC_1997–2000_ = −0.63%, APC_1997–2000_ = −1.60%), then shifted to an upward trend (APC_2007–2011_ = 0.61%), and finally reverted to a downward trend (APC_2011–2019_ = −1.30%). When analyzed by sex, similar to the global trend, the ASMR underwent three declines (APC_1990–1996_ = −1.39%, APC_1996–2007_ = −0.78%, and APC_2012–2019_ = −1.39%) and a brief increase midway (APC_2007–2012_ = 1.63%) among male. Meanwhile, the ASMR underwent six declines (APC_1990–1994_ = −2.33%, APC_1994–1997_ = −4.07%, APC_1997–2000_ = −1.30%, APC_2000–2007_ = −3.26%, APC_2007–2015_ = −2.43%, and APC_2015‐2019_ = −0.73%) among females.

**TABLE 2:** Joinpoint regression analysis of head and neck cancer in China from 1990 to 2019.

Variable	Both sexes			Male			Female		
	Segment	Period	APC (95% CI)	Segment	Period	APC (95% CI)	Segment	Period	APC (95% CI)
Overall	1	1990–1997	−2.0122 (−2.2005 to −1.8235)^a^	1	1990–1996	−1.3945 (−1.7275 to −1.0604)^a^	1	1990–1994	−2.328 (−3.0644 to −1.586)^a^
	2	1997–2000	−0.6253 (−1.7372 to 0.4992)	2	1996–2007	−0.7821 (−0.8957 to −0.6684)^a^	2	1994–1997	−4.0732 (−5.9752 to −2.1326)^a^
	3	2000–2007	−1.5956 (−1.7846 to −1.4063)^a^	3	2007–2012	1.6295 (1.1338 to 2.1276)^a^	3	1997–2000	−1.3008 (−3.0193 to 0.4481)^a^
	4	2007–2011	0.6071 (0.0393 to 1.1781)^a^	4	2012–2019	−1.387 (−1.6896 to −1.0834)^a^	4	2000–2007	−3.2578 (−3.5273 to −2.9875)^a^
	5	2011–2019	−1.2982 (−1.4738 to −1.1223)^a^	5	NA	NA	5	2007–2015	−2.4267 (−2.6484 to −2.2045)^a^
	6	NA	NA	6	NA	NA	6	2015–2019	−0.7281 (−1.5302 to 0.0807)
	AAPC	1990–2019	−1.2134 (−1.3616 to −1.065)^a^	AAPC	1990–2019	−0.6447 (−0.7742 to −0.5151)^a^	AAPC	1990–2019	−2.4383 (−2.7285 to −2.1472)^a^
LOC	1	1990–1999	−0.8913 (−1.133 to −0.649)^a^	1	1990–1998	−0.4945 (−0.6579 to −0.3308)^a^	1	1990–1993	−0.4377 (−1.3363 to 0.469)
	2	1999–2012	3.1298 (2.9776 to 3.2822)^a^	2	1998–2001	2.7324 (1.6004 to 3.8769)^a^	2	1993–1998	−2.0693 (−2.5143 to −1.6223)^a^
	3	2012–2019	−1.5304 (−2.0364 to −1.0217)^a^	3	2001–2004	5.8321 (4.5624 to 7.1172)^a^	3	1998–2011	−0.976 (−1.0495 to −0.9024)^a^
	4	NA	NA	4	2004–2007	3.9148 (2.7314 to 5.1119)^a^	4	2011–2015	−1.7886 (−2.4683 to −1.1042)^a^
	5	NA	NA	5	2007–2012	5.471 (5.0575 to 5.886)^a^	5	2015–2019	0.1242 (−0.5167 to 0.7694)
	6	NA	NA	6	2012–2019	−1.9818 (−2.2251 to −1.7379)^a^	6	NA	NA
	AAPC	1990–2019	0.734 (0.5819 to 0.8865)^a^	AAPC	1990–2019	1.5732 (1.3613 to 1.7856)^a^	AAPC	1990–2019	−1.0716 (−1.2378 to −0.9051)^a^
NPC	1	1990–1997	−2.4838 (−2.6455 to −2.3218)^a^	1	1990–1996	−1.9927 (−2.2315 to −1.7532)^a^	1	1990–1994	−2.6864 (−3.1497 to −2.2209)^a^
	2	1997–2000	−1.2732 (−2.23 to −0.307)^a^	2	1996–2000	−1.0337 (−1.5587 to −0.5058)^a^	2	1994–1997	−4.433 (−5.5862 to −3.2658)^a^
	3	2000–2007	−4.0516 (−4.2127 to −3.8902)^a^	3	2000–2007	−3.4168 (−3.5922 to −3.2411)^a^	3	1997–2000	−2.1442 (−3.1809 to −1.0965)^a^
	4	2007–2014	−2.2695 (−2.4437 to −2.0951)^a^	4	2007–2014	−1.5945 (−1.7888 to −1.3999)^a^	4	2000–2007	−5.7443 (−5.9022 to −5.5861)^a^
	5	2014–2019	−1.1074 (−1.4574 to −0.7561)^a^	5	2014–2019	−1.0972 (−1.4941 to −0.6988)^a^	5	2007–2014	−3.5683 (−3.7352 to −3.4012)^a^
	6	NA	NA	6	NA	NA	6	2014–2019	−1.0776 (−1.4199 to −0.7342)^a^
	AAPC	1990–2019	−2.4535 (−2.5777 to −2.3291)^a^	AAPC	1990–2019	−1.9577 (−2.0756 to −1.8397)^a^	AAPC	1990–2019	−3.4981 (−3.6713 to −3.3246)^a^
OPC	1	1990–1994	−0.1572 (−0.5857 to 0.2732)	1	1990–1995	−0.049 (−0.2831 to 0.1856)	1	1990–1993	−0.5983 (−2.2451 to 1.0761)
	2	1994–2000	−2.1568 (−2.3719 to −1.9412)^a^	2	1995–2000	−2.1243 (−2.3584 to −1.8897)^a^	2	1993–1998	−3.8766 (−4.6757 to −3.0709)^a^
	3	2000–2003	−2.7862 (−3.725 to −1.8383)^a^	3	2000–2003	−3.7492 (−4.4834 to −3.0092)^a^	3	1998–2011	−1.596 (−1.7285 to −1.4634)^a^
	4	2003–2007	−0.7615 (−1.2304 to −0.2905)^a^	4	2003–2006	−0.8825 (−1.6704 to −0.0883)^a^	4	2011–2015	−2.617 (−3.8481 to −1.3702)^a^
	5	2007–2010	0.971 (0.0308 to 1.92)^a^	5	2006–2011	1.2742 (1.031 to 1.5181)^a^	5	2015–2019	−0.7954 (−1.9825 to 0.4061)
	6	2010–2019	−0.4188 (−0.5474 to −0.29)^a^	6	2011–2019	0.0267 (−0.0988 to 0.1523)	6	NA	NA
	AAPC	1990–2019	−0.8968 (−1.0547 to −0.7386)^a^	AAPC	1990–2019	−0.638 (−0.7633 to −0.5125)^a^	AAPC	1990–2019	−1.9222 (−2.226 to −1.6175)^a^
LC	1	1990‐1997	−2.1254 (−2.3657 to −1.8845)^a^	1	1990–1997	−1.5608 (−1.767 to −1.3541)^a^	1	1990–1998	−4.6273 (−4.994 to −4.2592)^a^
	2	1997–2004	−0.3871 (−0.6316 to −0.142)^a^	2	1997–2000	0.0666 (−1.1202 to 1.2675)	2	1998–2004	0.9573 (0.3054 to 1.6135)^a^
	3	2004–2007	−3.0451 (−4.4079 to −1.6628)^a^	3	2000–2004	−1.2641 (−1.8816 to −0.6427)^a^	3	2004–2007	−4.2391 (−6.7421 to −1.669)^a^
	4	2007–2011	1.0103 (0.3011 to 1.7246)^a^	4	2004–2007	−2.4093 (−3.5616 to −1.2432)^a^	4	2007–2010	−0.1504 (−2.6813 to 2.4463)
	5	2011–2019	−1.1575 (−1.3831 to −0.9313)^a^	5	2007–2012	1.2743 (0.8884 to 1.6617)^a^	5	2010–2019	−2.2315 (−2.578 to −1.8838)^a^
	6	NA	NA	6	2012–2019	−1.1192 (−1.3567 to −0.881)^a^	6	NA	NA
	AAPC	1990–2019	−1.1084 (−1.2954 to −0.921)^a^	AAPC	1990–2019	−0.8503 (−1.0473 to −0.6528)^a^	AAPC	1990–2019	−2.2483 (−2.6383 to −1.8566)^a^
TC	1	1990–1998	−1.1704 (−1.3339 to −1.0066)^a^	1	1990–1994	−0.8983 (−1.7854 to −0.0032)^a^	1	1990–1998	−1.8713 (−2.0934 to −1.6487)^a^
	2	1998–2004	0.5556 (0.2868 to 0.825)^a^	2	1994–2001	1.2109 (0.8471 to 1.576)^a^	2	1998–2004	−0.995 (−1.3709 to −0.6177)^a^
	3	2004–2007	−0.1986 (−1.1288 to 0.7403)	3	2001–2007	3.1832 (2.8048 to 3.5631)^a^	3	2004–2015	−2.387 (−2.5142 to −2.2595)^a^
	4	2007–2011	2.4145 (1.9761 to 2.8548)^a^	4	2007–2012	6.1465 (5.4305 to 6.8673)^a^	4	2015–2019	−0.5576 (−1.2071 to 0.0962)
	5	2011–2019	−1.3904 (−1.5243 to −1.2563)^a^	5	2012–2019	−2.1082 (−2.5092 to −1.7056)^a^	5	NA	NA
	AAPC	1990–2019	−0.2875 (−0.4159 to −0.1588)^a^	AAPC	1990–2019	1.3374 (1.1236 to 1.5516)^a^	AAPC	1990–2019	−1.7068 (−1.8392 to −1.5742)^a^

Abbreviations: AAPC, average annual percentage change; APC, annual percentage change; CI, confidence interval; LC, larynx cancer; LOC, lip–oral cavity cancer; NA, not applicable; NPC, nasopharynx cancer; OPC, other pharynx cancer; TC, thyroid cancer.

^a^ Changes were statistically significant.

### 3.3 Age–Period–Cohort Analysis

#### 3.3.1 The Net Drift

We further calculated net drifts using the age–period–cohort analysis, as shown in Table S1. From 1990 to 2019, the overall age‐adjusted HNC mortality rates decreased by −1.62% (95% CI: −1.75, −1.49) per year. Moreover, the decline in mortality rates was significantly greater among females [−3.34% (95% CI: −3.55, −3.12)] than males [−1.37% (95% CI: −1.63, −1.10)]. When examining the data from each HNC anatomical site, NPC [−2.82% (95% CI: −2.95, −2.69)], OPC [−1.63% (95% CI: −1.86, −1.42)], LC [−1.84% (95% CI: −2.04, −1.64)], and TC [−0.63% (95% CI: −0.85, −0.41)] were consistent with the overall data, showing a decline. However, LOC [0.92% (95% CI: 0.76, 1.08)] demonstrated an opposing trend, with an increase observed.

#### 3.3.2 The Age Effects

**FIGURE 1:**
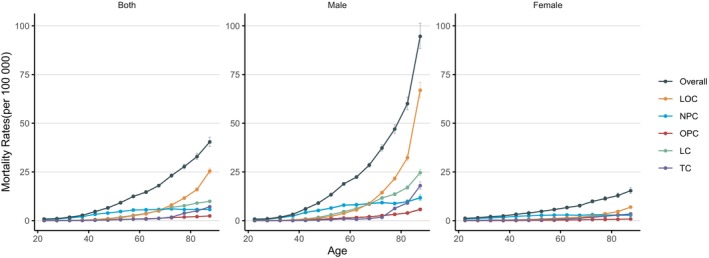
Longitudinal age curves of HNC mortality in China. Data provided for both sexes, male, and female. Fitted longitudinal age‐specific rates of HNC mortality (per 100 000). Error bars represent the 95% CIs for the longitudinal age curve values.

The longitudinal age curves of HNC mortality in China are illustrated in Figure 1. After adjusting for period effects and birth cohort effects, the mortality rate for HNC in China exhibits a monotonic increasing trend. The rate escalates from 0.76 per 100 000 (95% CI: 0.73, 0.95) in the 20–25 age group to 40.41 per 100 000 (95% CI: 38.09, 42.86) in the over 85 age group. The increase is more pronounced in males, rising from 0.40 per 100 000 (95% CI: 0.66, 0.89) in the 20–25 age group to 94.60 per 100 000 (95% CI: 88.38, 101.26) in the over 85 age group. Similarly, female HNC mortality rates also show an upward trend, but the increase is more gradual compared to males, moving from 1.20 per 100 000 (95% CI: 0.98, 1.47) in the 20–25 age group to 15.37 per 100 000 (95% CI: 13.94, 16.95) in the over 85 age group. Analyzing the data from various anatomical sites, the largest increase in mortality rates is observed in LOC, which surged from 0.05 per 100 000 (95% CI: 0.04, 0.06) to 25.41 per 100 000 (95% CI: 23.94, 26.99). Conversely, OPC exhibited the smallest growth, rising from 0.03 per 100 000 (95% CI: 0.03, 0.04) to 2.42 per 100 000 (95% CI: 2.21, 2.64).

#### 3.3.3 The Period Effects

**FIGURE 2:**
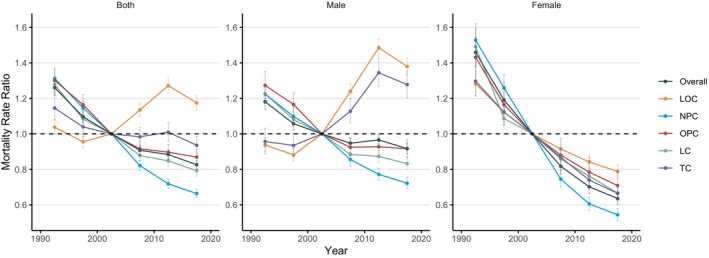
Period rate ratio (RR) of HNC mortality in China. Data provided for both sexes, male, and female. The RR of each period compared with the reference period (Years 2000–2005) adjusted for age and nonlinear cohort effects. Error bars represent the 95% CIs for the period RR.

Figure 2 shows the estimated period effects of the mortality of HNC in China. Using the period from 2000 to 2005 as a reference, the period effect on the mortality rate of HNC in China has been declining annually. The rate decreased from 1.26 per 100 000 (95% CI: 1.22, 1.30) in the period from 1990 to 1995 to 0.83 per 100 000 (95% CI: 0.80, 0.85) in the period from 2015 to 2019. The decline was less pronounced in males, from 1.18 per 100 000 (95% CI: 1.14, 1.23) to 0.92 per 100 000 (95% CI: 0.89, 0.95), and more substantial in females, from 1.46 per 100 000 (95% CI: 1.38, 1.54) to 0.64 per 100 000 (95% CI: 0.60, 0.67). When considering individual anatomical sites, NPC, OPC, and LC followed a similar downward trend as the overall tendency. In contrast, LOC and TC experienced an interim increase before returning to a downward trend in the 2010–2015 period.

#### 3.3.4 The Cohort Effects

**FIGURE 3:**
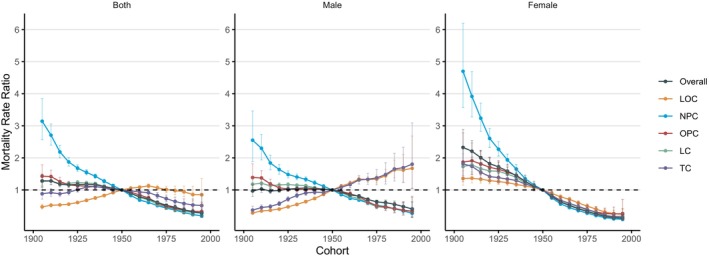
Cohort rate ratio (RR) of HNC mortality in China. Data provided for both sexes, male, and female. The RR of each cohort compared with the reference cohort (Cohort 1950) adjusted for age and nonlinear period effects. Error bars represent the 95% CIs for the cohort RR.

Figure 3 shows the estimated cohort effects of the mortality of HNC in China. Throughout the entire birth cohort, the cohort effect on the mortality rate of HNC exhibited minor fluctuations after reaching a peak of 1.28 (95% CI: 1.10, 1.49) in 1905, followed by a gradual decline starting in 1930, and reaching the lowest point of 0.28 (95% CI: 0.19, 0.41) in 1995. The cohort effect in males showed a similar pattern, with small fluctuations between 1905 and 1935, peaking at 1.06 (95% CI: 1.02, 1.11) in 1935, then slowly declining to the lowest point of 0.41 (95% CI: 0.27, 0.62) in 1995. In contrast, the cohort effect in females, although starting from a higher initial point, displayed a clear overall downward trend, decreasing from 2.33 (95% CI: 1.88, 2.88) in 1905 to 0.12 (95% CI: 0.06, 0.23) in 1995. When analyzed by different anatomical sites, the most significant decline was observed in NPC, which decreased from 3.14 (95% CI: 2.56, 3.85) in 1905 to 0.19 (95% CI: 0.13, 0.28) in 1995. The trends for OPC, LC, and TC were not markedly different from the overall trend. However, LOC showed a notable upward trend from 1905 to 1965, reaching a peak of 1.13 (95% CI: 1.07, 1.19) in 1965, before slowly declining to the lowest point of 0.85 (95% CI: 0.53, 1.36) in 1995.

## 4 Discussion

This study found a significant decline in HNC mortality rates in China from 1990 to 2019, with an AAPC of −1.21%. The decline was more pronounced in females (−3.34% per year) compared to males (−1.37%). Among specific cancer types, NPC, OPC, LC, and TC showed decreasing trends, whereas LOC exhibited an increasing trend. JPR analysis revealed a brief upward trend between 2007 and 2011, followed by a return to decline. These findings emphasize the importance of continued efforts in HNC prevention and treatment, particularly for females and the increasing LOC trend.

The incidence and mortality rates of cancer worldwide are rapidly increasing, largely due to factors such as population aging and growth, changes in the prevalence and distribution of major cancer risk factors, and socioeconomic development [6]. As a major type of cancer, the incidence of HNC has been rising over recent decades [2]. Global demographic and epidemiological trends indicate that the incidence of HNC will continue to rise in the coming decades [6]. The Global Cancer Report (GLOBOCAN 2020) revealed that in 2020, HNC ranked eighth in global incidence and twelfth in mortality [6]. The findings of this study show that in 2019, the global ASMR for HNC was 6.74 per 100 000, with the ASMR for males at 10.18 per 100 000 and for females at 3.67 per 100 000. In China, the ASMR for HNC was 4.27 per 100 000, with males at 6.98 per 100 000 and females at 1.96 per 100 000, which is still at a relatively high level globally, indicating that the mortality burden of HNC in China remains severe. Moreover, the study found that although the ASMR for HNC in China generally showed a downward trend from 1990 to 2019, some anatomical subtypes (such as LOC) are still on the rise. Therefore, understanding the mortality trends of HNC and their anatomical subtypes is of great practical significance for reducing the disease burden of HNC and controlling their progression in China.

As the population ages, the risk of mortality from HNC in China gradually increases. This may be associated with the reduced treatment tolerance, multiple comorbidities, altered pharmacokinetics and pharmacodynamics [27, 28], as well as the intensification of population aging in China. Data from the National Bureau of Statistics indicates that since 1978, the number and proportion of the elderly population aged 65 and above in China have been continuously increasing. From 2001 to 2018, the annual growth rate of the elderly population aged 65 and above was 3.28%, significantly higher than the total population's annual growth rate of 0.66% [29]. The acceleration of population aging leads to an increased risk of mortality and disease burden from HNC.

The less pronounced decline in mortality among males compared to females in China may suggest differences in exposure to risk factors, access to healthcare, or biological factors that warrant further investigation. It is known that lifestyle factors such as smoking and alcohol consumption, which are more prevalent among males, are significant risk factors for HNC [30, 31, 32]. Meanwhile, the lower female HNC risk may, in part, be explained by endogenous and exogenous estrogen exposures [33].

When considering individual anatomical sites, the downward trend in NPC, OPC, and LC mortality rates is consistent with the overall tendency. This could be attributed to targeted public health interventions and advances in treatment specific to these cancers. For instance, the implementation of NPC screening programs in high‐incidence areas and the introduction of HPV vaccination, which is associated with a subset of OPC, may have contributed to these trends [34, 35, 36]. In contrast, the interim increase in mortality rates for LOC and TC before returning to a downward trend in the 2010–2015 period could reflect changes in environmental factors, healthcare policies, or diagnostic practices over time. The increase might also be due to the lag effect of exposure to risk factors (such as betel chewing [37, 38]) or variations in the adoption of improved clinical guidelines across different regions of China [39].

This study provides valuable insights into the long‐term mortality trends of HNC in China, utilizing nearly 30 years of data from the GBD 2019 database. One of the key strengths of this study is its comprehensive approach, combining JPR and age–period–cohort effect analysis to reveal the temporal dynamics of HNC mortality, as well as the differences across sex and anatomical subtypes. The novelty of this study lies in its focus on both the overall trend and the specific anatomical subtypes of HNC, offering a more detailed understanding of the disease burden in China.

The methodology employed in this study aligns with recent research [40], which similarly utilized JPR and age–period–cohort analysis to assess cancer mortality trends. This approach is well‐suited for analyzing complex temporal trends and providing a nuanced understanding of changes in disease burden across different time periods and demographic groups. By incorporating both JPR and age–period–cohort effects, our study builds on these established methods while offering more granular insights into HNC trends in China, particularly with respect to gender and specific cancer subtypes.

Despite these strengths, the study has several limitations. First, it is an observational, cross‐sectional analysis that does not directly investigate the underlying causes of the observed trends, such as changes in risk factors, healthcare access, or clinical practices. Additionally, the use of the GBD database, while valuable for providing large‐scale population‐level data, has some inherent limitations. The mortality rate coding for HNC in the GBD database includes some ICD‐10 codes that represent benign diseases. Given that benign diseases should not be the direct cause of death, this study leans toward the possibility that they may have been misclassified, as a previous publication mentioned [41]. Furthermore, the lack of distinction between urban and rural mortality data within China may obscure regional differences that could be important for more targeted public health interventions. Future studies would benefit from more granular, region‐specific data and a deeper investigation into the factors influencing HNC mortality trends in different demographic groups.

## 5 Conclusion

In summary, from 1990 to 2019, the ASMR for HNC in China generally showed a downward trend, with the risk of death increasing with age and over time, and those born earlier facing a greater risk of death. The increasingly prominent issue of population aging in China may further exacerbate the disease burden of HNC in the future. Therefore, further efforts should be made to strengthen early diagnosis and treatment, find more effective treatment methods for HNC, and reduce the disease burden. Enhancing the collection and organization of cancer registry data for HNC in China, especially for a comprehensive understanding of different anatomical subtypes of HNC, is of great significance for further reducing the disease burden of HNC in China.

## Author Contributions

**Zhengxin Zhu:** conceptualization (lead), data curation (lead), formal analysis (lead), investigation (lead), methodology (lead), resources (supporting), software (supporting), validation (supporting), visualization (supporting), writing – original draft (lead), writing – review and editing (lead). **Jiasheng Yuan:** conceptualization (supporting), data curation (supporting), investigation (supporting), software (supporting), writing – original draft (supporting). **Li Fu:** data curation (supporting), investigation (supporting), methodology (supporting), resources (supporting), writing – review and editing (supporting). **Wanqing Zhang:** formal analysis (supporting), validation (supporting), writing – review and editing (supporting). **Songtao Liu:** formal analysis (supporting), visualization (supporting), writing – review and editing (supporting). **Yuehui Liu:** conceptualization (supporting), methodology (supporting), project administration (lead), resources (supporting), supervision (lead), writing – review and editing (supporting).

## Ethics Statement

The authors have nothing to report.

## Conflicts of Interest

The authors declare no conflicts of interest.

## Supporting information


**Table S1.** Net drift for head and neck cancer from 1990 to 2019.

## Data Availability

The authors have nothing to report.
